# Supplementary material for: Kinase Inhibitor Treatment of Patients with Advanced Cancer Results in High Tumor Drug Concentrations and in Specific Alterations of the Tumor Phosphoproteome
Source: Cancers (Basel). 2020 Feb 1;12(2):330. doi: 10.3390/cancers12020330 (PMC7072422; doi:10.3390/cancers12020330)
Supplement: Supplementary file 1 [file cancers-12-00330-s001.zip › cancers-680478-supplementary-final/cancers-680478-supplement-final.pdf]

Supplementary Material

Kinase Inhibitor Treatment of Patients with Advanced Cancer Results in High Tumor Drug Concentrations and in Specific Alterations of the Tumor Phosphoproteome

Mariette Labots, Thang V. Pham, Richard J. Honeywell, Jaco C. Knol, Robin Beekhof, Richard de Goeij-de Haas, Henk Dekker, Maarten Neerincx, Sander R. Piersma, Johannes C. van der Mijn, Donald L. van der Peet, Martijn R. Meijerink, Godefridus J. Peters, Nicole C.T. van Grieken, Connie R. Jiménez and Henk M.W. Verheul

Table S1. PKI concentration in tissue and blood from individual patients

Table S2. Metrics of obtained protein and identified phosphopeptides and -sites per patient

Table S3. All identified and quantified phosphopeptides *

Table S4. All identified phosphosites and sample labeling *

Table S5. List of highly regulated phosphopeptides in at least 3 patients per cohort *

Table S6. PKI target profiles

Figure S1. Patient disposition flowchart

Figure S2. Pearson's correlations of HCT 116 control samples

Figure S3. Protein interaction networks of PKI-regulated phosphopeptides

Figure S4. Protein interaction networks of PKI-regulated phosphopeptides related to tumor concentration

Figure S5. Example of substrate inhibition in relation to sorafenib response

* Due to file size, Table S3, S4 and S5 (.xls) could not be included in this .docx file but have been uploaded separately.

Table S1. PKI concentrations in tissue and blood from individual patients.

Patient ID	Tumor type	Tumor	Skin	Plasma	Serum
SOR 1	Hepatocellular	6.9	4.4	8.5	11.6
SOR 2	Melanoma	22.0	7.2	4.2	6.9
SOR 3	Hepatocellular	10.0	6.3	4.8	6.1
SOR 4	Hepatocellular	3.7	1.4	3.7	4.8
SOR 5	Thyroid, papillary	22.9	28.4	12.1	17.4
ERL 1	Pancreatic	10.8	2.1	1.9	2.1
ERL 2	HNSCC	0.9	2.3	1.2	1.0
ERL 3	Pancreatic	8.8	6.7	4.0	4.4
ERL 4	Rectal	1.7	2.8	0.9	0.9
ERL 5	Esophageal	4.2	3.3	1.1	1.1
DAS 1	Esophageal	18.9	0.4	0.016	0.017
DAS 2	Colorectal	2.0	NA	0.041	0.037
DAS 3	Prostate	64.0	18.5	0.005	No peak
DAS 4	Colorectal	0.2	0.3	0.012	No peak
DAS 5	Melanoma	0.5	0.2	0.007	0.009
VEM 1	Melanoma	2347.3	391.9	98.2	46.7
VEM 2	Melanoma	1192.0	2449.4	94.6	119.7
VEM 3	Melanoma	330.7	749.1	108.0	131.0
VEM 4	Melanoma	1459.4	2016.6	209.7	190.0
VEM 5	Melanoma	1016.5	957.3	192.7	241.9
VEM 6	Melanoma	1630.9	2557.1	65.0	75.3
VEM a	Melanoma	NA	800.1	81.4	97.1
VEM b	Melanoma	NA	119.6	NA	48.6
SUN 1	Clear cell sarcoma	2.4	NA	0.14	0.14
SUN 2	ACUP	2.3	9.7	0.16	0.16
SUN 3	Colorectal	13.7	NA	0.11	0.12
SUN 4	Colorectal	9.0	0.5	0.08	0.09
SUN 5	Renal cell	50.0	4.3	0.11	0.12
EVE 1	Renal cell	3.6	NE	NE	NE
EVE 2	Renal cell	3.4	NE	NE	NE
EVE 3	Renal cell	NE	NE	NE	NE
EVE 4	Pancreatic NET	NE	NE	NE	NE
EVE 5	NET	NE	NE	NE	NE

Concentrations in μM after 10–14 days in patients for whom on-treatment biopsies were available for evaluation of tumor concentration and/or phosphoproteomics. *VEM a,b*; Additional skin biopsies were obtained from 2 patients in whom no on-treatment tumor tissue was available after treatment. In VEM a, tumor re-biopsy was not possible due to response of the lesion. On-treatment tumor biopsy was not deemed safe for VEM b. NA; not available. NE; not evaluable. .

Table S2. Metrics of obtained protein and identified phosphopeptides and -sites per patient.

Patient ID	Needle size (G)	Protein yield / biopsy (µg)		Protein input (mg)	# Phospho-peptides		# Phosphosites		Phosphosite enrichment					
		Pre-tx	On-tx	Pre- & On-tx	Pre-tx	On-tx	Pre-tx	On-tx	%S (pre - on)		%T (pre - on)		%Y (pre-on)	
**SOR 1**	14 - 14	2990	3050	2.9	506	469	414	373	2,9	3,0	2,4	2,1	94,7	94,9
**SOR 2**	14 - 14	2240	7660	2.0	386	384	304	297	1,3	1,7	3,3	2,4	95,4	96,0
**SOR 3**	14 - 14	2760	1490	1.5	412	421	308	329	2,0	1,5	2,9	3,3	95,1	95,1
**SOR 4**	14 - 14	3080	2270	2.0	461	436	361	343	2,5	2,9	3,1	3,2	94,5	93,9
**SOR 5**	16 - 14	930	1560	1.0	342	371	262	286	2,3	2,8	3,4	3,2	94,3	94,1
**ERL 1**	14 - 14	1280	950	1.0	404	439	330	348	7,6	7,8	3,9	4,3	88,5	87,9
**ERL 2**	14 - 14	2780	2770	2.5	639	621	505	495	2,6	3,2	4,0	4,0	93,5	92,7
**ERL 3**	14 - 16	2990	2390	2.2	653	434	530	372	5,3	4,3	3,8	4,3	90,9	91,4
**ERL 4**	14 - 14	3290	2330	2.2	435	701	346	584	2,3	4,3	2,9	3,6	94,8	92,1
**ERL 5**	18 - 16	1160	1240	1.1	545	465	440	369	3,0	2,4	4,6	2,4	92,5	95,1
**DAS 1**	GI - GI	1560	1530	1.5	687	598	556	477	3,6	2,7	3,4	3,6	93,0	93,7
**DAS 2**	14 - 16	5170	1990	2.0	555	745	450	590	3,1	3,6	2,7	2,0	94,2	94,4
**DAS 3**	14 - 14	3160	2050	2.0	668	659	545	513	3,3	2,5	3,1	3,1	93,6	94,4
**DAS 4**	16 - 16	580	1000	0.6	292	316	245	269	2,9	4,8	3,7	3,0	93,5	92,2
**DAS 5**	14 - 14	2060	2640	2.0	548	516	434	432	1,8	1,6	3,0	3,2	95,2	95,1
**VEM 1**	NA - 14	NA	>3000	1.5*	225	193	175	151	1,7	1,3	3,4	3,3	94,9	95,4
**VEM 2**	14 - 14	620	360	0.6	251	NE	193	NE	0,5	NE	3,6	NE	95,9	NE
**VEM 3**	18 - 18	660	960	0.7 – 0.9	129	193	98	163	2,0	1,2	5,1	4,9	92,9	93,9
**VEM 4**	14 - 14	1360	1750	1.3	177	294	148	231	2,7	0,9	4,1	3,9	93,2	95,2
**VEM 5**	14 - 14	1620	990	1.0	201	197	156	155	3,2	3,2	1,9	4,5	94,9	92,3
**VEM 6**	14 - 14	1710	1050	1.1	243	211	190	167	1,6	3,6	2,6	3,0	95,8	93,4

Patient ID	Needle size (G)	Protein yield / biopsy (µg)		Protein input (mg)	# Phospho-peptides		# Phosphosites		Phosphosite enrichment					
		Pre-tx	On-tx	Pre- & On-tx	Pre-tx	On-tx	Pre-tx	On-tx	%S (pre - on)		%T (pre - on)		%Y (pre-on)	
**SUN 1**	16 - 16	590	790	0.6	97	89	85	71	1,2	4,2	5,9	2,8	92,9	93,0
**SUN 2**	14 - 14	2340	2530	2.0	458	507	369	414	2,4	4,4	4,1	4,3	93,5	90,8
**SUN 3**	16 - 14	450	2860	0.45<br>[2]**	146	264<br>[491]	123	202<br>[403]	3,3	2,5<br>[3,0]	2,4	5,0<br>[4,7]	94,3	92,6<br>[92,3]
**SUN 4**	16 - 16	500	530	0.5	446	325	375	268	3,2	3,7	4,5	3,0	92,3	93,3
**SUN 5**	14 - 14	4990	340	0.35<br>[2]***	149<br>[378/441]	NE	116<br>[306/352]	NE	3,5<br>[3,3/3,1]	NE	1,7<br>[1,6/2,0]	NE	94,8<br>[95,1/94,9]	NE
**EVE 1**	14 - 14	2697	1208	1.2	NE	NE	NE	NE	NE	NE	NE	NE	NE	NE
**EVE 2**	18 - 18	441	NA	0.4	NE	NE	NE	NE	NE	NE	NE	NE	NE	NE
**EVE 3**	14 - 14	728	1530	0.7	NE	NE	NE	NE	NE	NE	NE	NE	NE	NE
**EVE 4**	14 - 14	3389	2394	2.3	NE	334	NE	252	NE	2,4	NE	2,4	NE	95,2
**EVE 5**	18 - 16	679	849	0.7	28	189	30	138	13,3	3,6	3,3	3,6	83,3	92,8

Protein yield, protein input, identified number (#) of phosphopeptides and phosphosites are shown per pre- and on-treatment (tx) biopsy of individual patients in each PKI cohort. Phosphosite enrichment: percentage phosphorylation on serine (S), threonine (T) and tyrosine (Y) residues of total number identified phosphosites. *G*, gauge; *NE*, not evaluable. Protein input was matched for pre- and on-treatment biopsies per individual patients, with the exception of patient VEM 3. *, results of VEM 1 are from 2 workflow replicates of on-treatment biopsy. ** For SUN 3, an additional on-treatment sample of 2 mg was profiled (SUN 3 on2_CRC in Figure 2A). *** For SUN 5, an additional pre-treatment duplo of 2 mg was profiled..

Table S3: All identified and quantified phosphopeptides

Table S4. All identified phosphosites and sample labeling

Table S5. List of highly regulated phosphopeptides in at least 3 patients per cohort

Table S6. PKI target profiles.

Drug	Targets
Sunitinib	VEGFR1-3, PDGFRα-β, KIT, FLT-3, CSF-1R, RET
Sorafenib	VEGFR2-3, B-raf, PDGFRα-β, KIT, RET
Dasatinib	BCR-ABL, SRC family (SRC, LCK, YES, FYN), KIT, EPHA2, PDGFRβ
Erlotinib	EGFR
Vemurafenib	V600E mutated BRAF
Everolimus	mTOR

Listed targets for the 6 applied drugs in this study were derived from the PubChem https://pubchem.ncbi.nlm.nih.gov/ and Drugbank databases https://pubchem.ncbi.nlm.nih.gov/source/DrugBank.

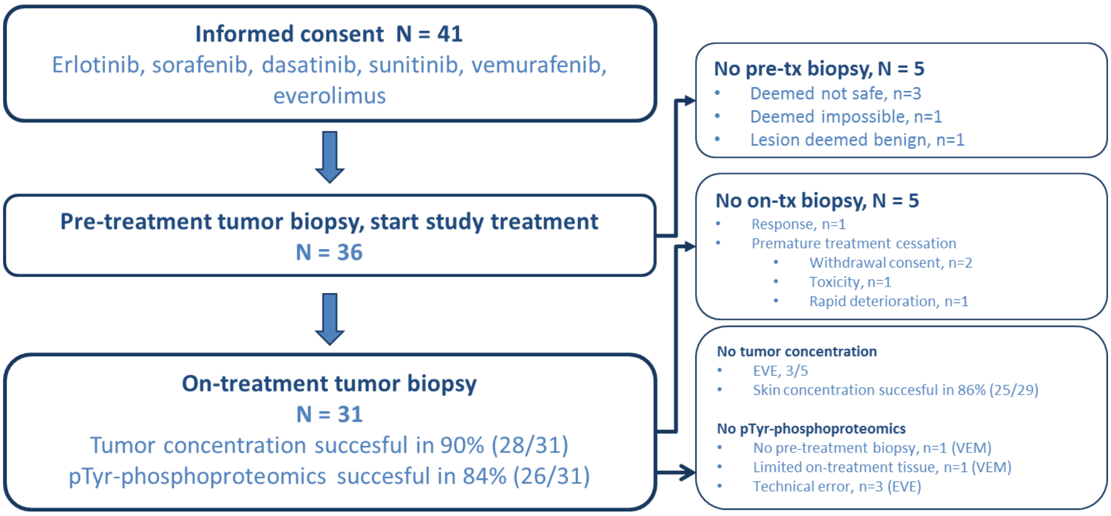

Figure S1. Patient disposition flowchart. Forty-one patients signed informed consent. Six cohorts were completed, i.e., with 5 evaluable patients defined as successfully performed pre- and on-treatment tumor biopsy. Five of 41 patients did not start study treatment due to the impossibility of a pre-treatment tumor biopsy based on the judgement of the interventional radiologist. Tumor re-biopsy was performed in 86% (31/36) of patients whom initiated study treatment; these patients were evaluable for the primary endpoint.

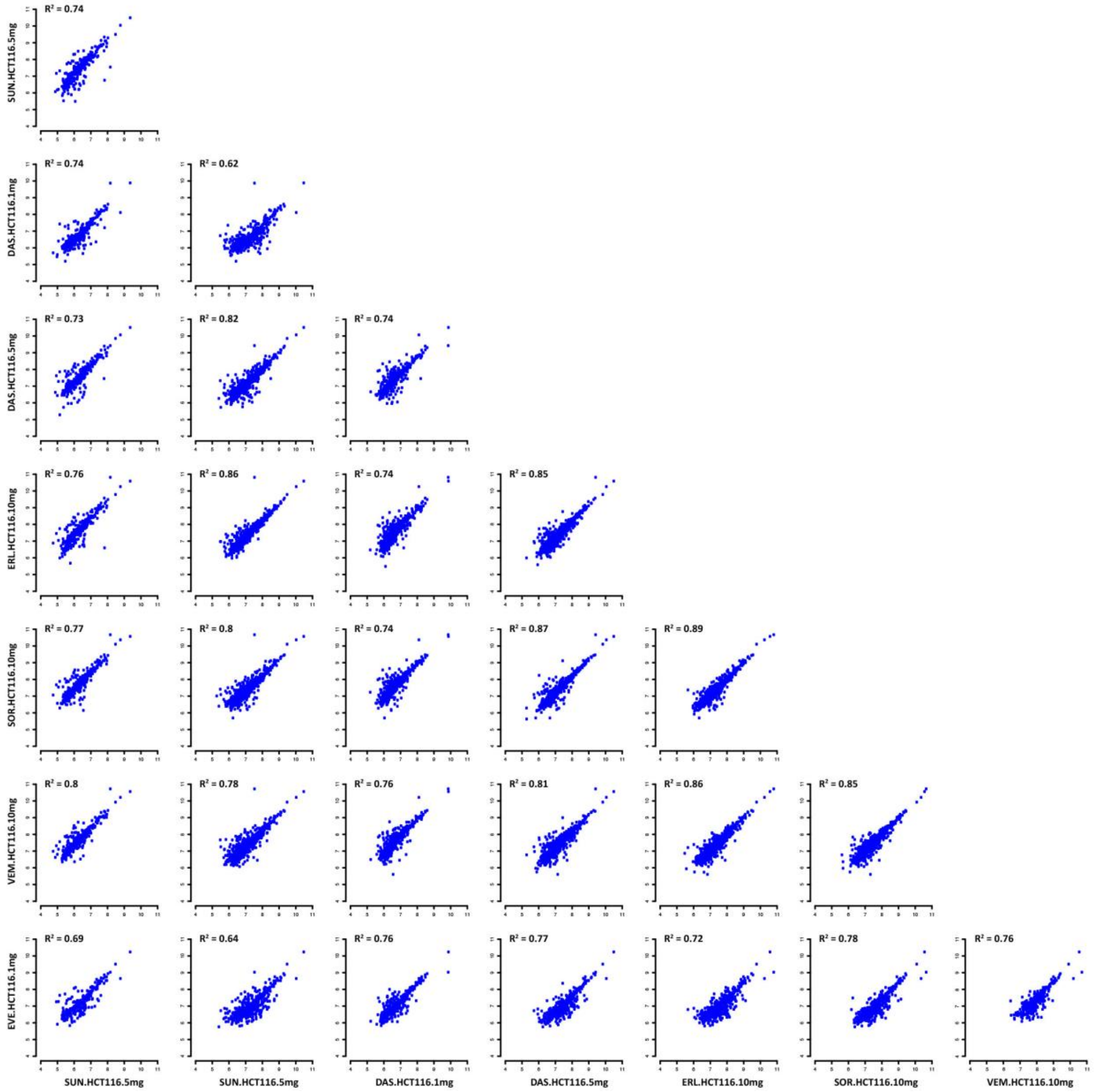

Figure S2. Pearson's correlations of HCT 116 control samples. Analysis of Pearson's correlations between control samples using 1, 5 or 10 mg lysate protein from colorectal cancer cell line HCT116 (measured along with the SUN, DAS, ERL, SOR, VEM and EVE drug cohorts on subsequent days) indicated that the differences in regulated peptides between the drug cohorts could not be attributed to day to day variation or batch effects.

.

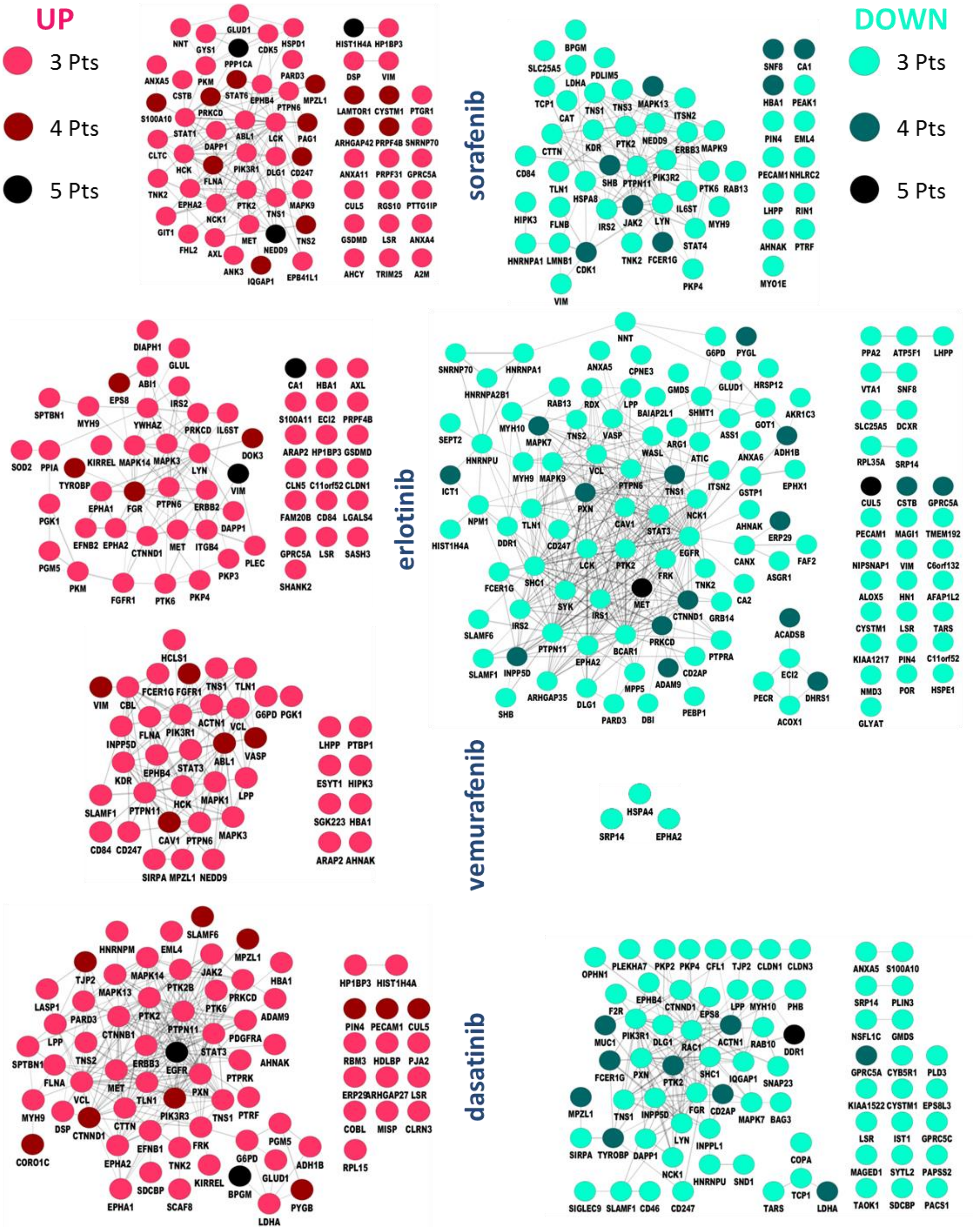

Figure S3. Protein interaction networks of PKI-regulated phosphopeptides. Protein interaction networks of Fc > 1.5 upregulated (red) and downregulated (green) phosphopeptides in ≥ 3 patients per cohort. Color indicates number of patients per cohort in which the regulation was observed (VEM, 4 patients; SOR/ERL/DAS, 5 patients; SUN not shown).

3 Pts

4 Pts

5 Pts

UP Fc > 1.5

DOWN Fc < -1.5

3 Pts

4 Pts

5 Pts

sorafenib

erlotinib

dasatinib

vemurafenib

Attached Peptides Correlating with Drug Concentration

Figure S4. Protein interaction networks of PKI-regulated phosphopeptides related to tumor concentration. Protein interaction networks of Fc > 1.5 upregulated (red) and downregulated (green) phosphopeptides in ≥ 3 patients per cohort. Color indicates number of patients per cohort in which the regulation was observed (*VEM*, 4 patients; *SOR*/*ERL*/*DAS*, 5 patients; *SUN* not shown).

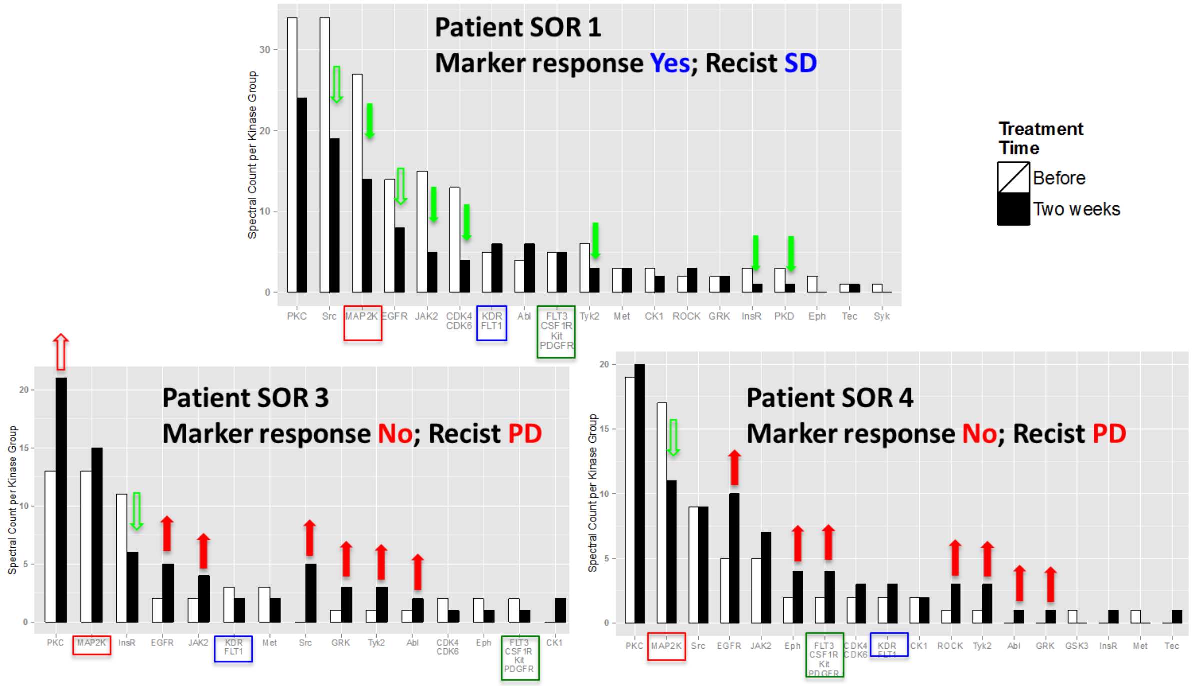

Sorafenib targets: MEK (red), VEGFR and KDR (blue) and KIT, FLT3 and PDGFR (green)

Figure S5. Example of substrate inhibition in relation to sorafenib response. NetworKIN analysis of kinase activities observed in pre- (white) and on-treatment (black) tumor biopsies in 3 patients with metastatic hepatocellular carcinoma treated with sorafenib. Patient SOR 1 had a CA 19-9 marker response and prolonged stable disease upon treatment, whereas patients SOR 3 and SOR 4 exhibited primary progressive disease.
